# The Multiplicity of Medical Societies

**Published:** 1888-05

**Authors:** 


### EDITORIAL.

### THE MULTIPLICITY OF MEDICAL SOCIETIES.

When the American Medical Association was organized, forty years ago, the object of its originators was, undoubtedly, to fraternize the members of the profession in the United States by annual meetings, and to add to the general store of medical knowledge by a conference of the medical men of the whole country.

Why this worthy and desirable object has not been fully attained is evident. At the time of the organization of the association the undeveloped condition of the country, the great distances necessary to be traveled in order to attend its annual meetings, held at different points, made it difficult for many to be present.

Local societies were developed to accommodate those who could not attend the meetings of the association.

Then, about twenty years ago, the tendency to the formation of specialties was developed, and from that time has run into an extreme. Again, local societies were organized to furnish these specialists fields in which to discuss their theories. Thus, city, village, county, district, and State medical societies multiplied, until now the number is so great, the times and places of meeting so many, that it is difficult for one to remember even the names of such organizations. The number of such general societies of regular physicians, published in a recent medical and surgical directory of the United States (1886), is 648. To these must also be added more national societies of specialties.

These societies have, therefore, diverted many contributions of knowledge from their proper repository — the American Medical Association. From being a source of only good, they have interfered more or less with the object of the parent organization, through their tendency to separate too much the departments of medicine. The growing tendency of recent years to drifting apart—to isolation—on the part of specialists is not in their interest, and is to be deprecated for many reasons.

We recognize the advantage of the division of labor for those qualified for special work. The contributions to the science of medicine by the qualified workers has given prominence to specialists, and tempted younger, unqualified men to attempt special fields of labor, and to organize more special societies. Limited professional experience has led many to ignore the advantage of a broad, liberal education, and many have acknowledged that they know but little outside of their special field, thus practically separating themselves from the profession. What began as an advantageous principle has been abused in many instances, and this tendency to isolation has been, in some respects, a detriment to the profession by the holding aloof of many able specialists from the American Medical Association, and thus interfering with her attempts to foster and cultivate these special fields, and materially diminishing the contributions from those fields of study that should reach the profession through that organization, which, more fully than any other, represents the whole profession of America.

Although mistakes have been made by the parent institution, as all will admit, it is evident to an unprejudiced observer that the desire is to adhere to its original object in its formation. The growth of the institution embarrasses it in carrying out the original plan; but its faults are not insuperable, and if those who have become alienated from it, and established or identified themselves with other organizations that semingly are conflicting with its interests, were to manifest the same interest and zeal in promoting the welfare of the association, by attending its meetings and contributing to its scientific work, many of its mistakes would be corrected, and an identity of interest of the medical profession be maintained.

It is now recognized that our civil war was largely the outgrowth of the want of closer intercourse and better acquaintance between the different sections of the country. It is not impossible that the same factors have been active in producing some of the existing alienations from the American Medical Association. It is much to be desired that the next meeting of the association, at Cincinnati, this year, may show the existence of better feeling.

